# The Use of the Health of the Nation Outcome Scales for Assessing Functional Change in Treatment Outcome Monitoring of Patients with Chronic Schizophrenia

**DOI:** 10.3389/fpubh.2016.00220

**Published:** 2016-10-13

**Authors:** Stephan T. Egger, Stefan Vetter, Godehard Weniger, Caroline Vandeleur, Erich Seifritz, Mario Müller

**Affiliations:** ^1^Department of Psychiatry, Psychotherapy and Psychosomatics, University Hospital of Psychiatry, Zurich, Switzerland; ^2^Department of Psychiatry, University Hospital of Lausanne, Lausanne, Switzerland

**Keywords:** schizophrenia, functioning, treatment outcome, Health of the Nation Outcome Scales, Positive and Negative Symptom Scales

## Abstract

**Background:**

Schizophrenia is a severe mental disorder that is characterized not only by symptomatic severity but also by high levels of functional impairment. An evaluation of clinical outcome in treatment of schizophrenia should therefore target not only assessing symptom change but also alterations in functioning. This study aimed to investigate whether there is an agreement between functional- and symptom-based outcomes in a clinical sample of admissions with chronic forms of schizophrenia.

**Methods:**

A full 3-year cohort of consecutive inpatient admissions for schizophrenia (*N* = 205) was clinically rated with the Positive and Negative Symptom Scale (PANSS) and the Health of the Nation Outcome Scales (HoNOS) as measures of functioning at the time of admission and discharge. The sample was stratified twofold: first, according to the degree of PANSS symptom improvement during treatment with the sample being divided into three treatment response groups: non-response, low response, and high response. Second, achievement of remission was defined using the Remission in Schizophrenia Working Group criteria based on selected PANSS symptoms. Repeated measures analyses were used to compare the change of HoNOS scores over time across groups.

**Results:**

More than a half of all admissions achieved a symptom reduction of at least 20% during treatment and around one quarter achieved remission at discharge. Similarly, HoNOS scores improved significantly between admission and discharge. Interaction analyses indicated higher functional improvements to be associated with increasing levels of treatment response.

**Conclusion:**

Functional improvement in individuals treated for schizophrenia was linked to a better clinical outcome, which implies a functional association. Thus, improvement of functioning represents an important therapeutic target in the treatment of schizophrenia.

## Introduction

The Health of the Nation Outcome Scales (HoNOS) (1, 2) have gained increasing attention as general measures for treatment outcome in psychiatric settings (3). The HoNOS were developed to provide brief, accurate, and relevant measures of overall mental health and social functioning for clinical practice (1). A growing body of evidence suggests that the HoNOS are related to illness severity (4, 5), overall clinical pre–post change (4, 6), overall symptom improvement, and a history of treatment utilization (7), as well as the length of hospital stay (8). However, the HoNOS seem to perform differently across the diagnostic spectrum (9, 10). In fact, earlier findings from our study showed that schizophrenic psychotic disorders, compared to other common mental disorders, were related to poorer HoNOS baseline levels (11), as well as to only marginal change over time (12). Despite an over-representation of chronic and schizophrenia patients in the development and validation studies of the HoNOS (13), evidence for the usefulness of the HoNOS as a measure for functional change in outcome monitoring of schizophrenic patients in relation to psychosis-specific measures is still rare.

In many aspects, schizophrenia can be described as a poor outcome disorder (14). So far, there is no universally accepted definition of remission for schizophrenia; but, however, there is a broad consensus that not only symptom improvement but also a reduction of functional impairment should be considered (15, 16). Actually, it can be assumed that symptomatic and functional improvement is functionally related to patients with schizophrenia (17). An operationalization of remission was proposed by the Remission in Schizophrenia Working Group (RSWG) (18) based on temporal and severity criteria. Although this definition has been used in a number of clinical trials, level and course of functioning were not part of this definition.

Therefore, the current study aimed to fill this gap by exploring whether the level of symptomatic treatment response between admission and discharge as well as remission status was systematically linked to change of functioning in a sample of inpatients with chronic schizophrenia. For this purpose, we examined whether either the level of symptomatic improvement (treatment response) or meeting remission criteria according to the Positive and Negative Symptom Scale (PANSS) was linked to an overall functional improvement as measured by the HoNOS.

## Materials and Methods

### Sample and Procedure

The Center for Integrative Psychiatry [ZIP (German): Zentrum für Integrative Psychiatrie], as part of the Psychiatric University Hospital of Zurich, is a specialist unit for the treatment of “heavy-users,” i.e., those patients with frequent and/or long-term hospitalizations for whom outpatient treatment is often insufficient to prevent relapse (19, 20).

Our study sample consists of a full 3-year cohort of consecutive inpatient admissions referred to our center for treatment between June 1, 2012 and June 30, 2015. All patients who were diagnosed with a schizophrenic psychosis according to the WHO ICD-10 (21) (*N* = 216) diagnostic criteria were considered for the current study. Patients with treatment duration shorter than 7 days (*N* = 11) were excluded to avoid overlap since ratings at discharge require a minimal retrospective observation period of 7 days.

Treatment followed an integrative approach in accordance with current treatment guidelines, including psychopharmacological and psychotherapeutic treatment, as well as psychosocial interventions (22–24). Treatment programs were individually adapted to the patients’ symptomatic load and level of functional impairment. The study was approved by the local Ethics Committee of the Canton of Zurich, Switzerland.

### Raters and Training

Raters were clinicians, i.e., either psychiatrists or psychiatry residents or clinical psychologists. All raters were trained in specific workshops on the use and objectives of the measures used in the study. The workshops followed a standardized schedule, using case vignettes and video examples. Refresher training sessions were provided on a regular basis, at least twice a year, with trainers being available for consultation at any time. On all measures, information was rated retrospectively for the 7 days prior to admission and again for the 7 days prior to discharge. All relevant information was derived from either the admission and discharge interviews or directly by behavioral observation, while additional information was provided by the nursing staff, social workers, and significantly others.

### Measures

#### Positive and Negative Symptom Scale

The PANSS is a semistructured interview that was designed to measure symptom severity in individuals with a psychotic disorder, in particular schizophrenia and schizoaffective disorder (25, 26). The PANSS consists of 30 items with 3 subscales for the domains of positive (7 items), negative (7 items), and non-specific symptoms (16 items), although only the total score was considered for the current study. Each symptom item is rated on a 7-point Likert scale response format from 1 (non-present) to 7 (very severe), with a possible range from 30 to 210 (25).

#### Treatment Response

Treatment response was defined by the degree of symptom improvement according to the PANSS, following the criteria used in psychopharmacological trials (27, 28), with a symptom reduction of 20 or 50% of the initial PANSS score used as a cutoff to define response (28–30). In order to obtain an appropriate measure of relative change, the PANSS was transformed beforehand into a ratio scale by subtracting 30 points from the total score (31). Accordingly, the study sample was assigned into three groups of treatment response: (1) high response (HR): those patients who had a ≥50% reduction of the PANSS total score between admission and discharge; (2) low response (LR): those patients who had a reduction between 20 and 49% of the PANSS total score; and (3) non-response (NR): those patients who showed a change lower than 20%.

#### Remission Status

Patients were classified as remitted (REM) according to the Remission in Schizophrenia Working Group criteria (RSWGcr) (18), when all of the following eight PANSS items, delusions, conceptual disorganization, hallucinatory behavior, blunted affect, social withdrawal, lack of spontaneity, mannerisms posturing, and unusual thought content, were rated as mild or less severe (i.e., score ≤3 points) at discharge. Otherwise, a patient was classified as non-remitted (NoREM) (18).

#### Health of the Nation Outcome Scales

The HoNOS are observer-rated scales and comprise 12 domains of functioning (aggressiveness; non-accidental self-injury; problem drinking or drug taking; cognitive problems; physical illness or disability; hallucinations and delusions; depressed mood; other mental and/or behavioral problems; problems with relationships; problems with activities of daily living; problems with living conditions; and problems with occupation and activities). Each scale can be rated from 0 (no problem) to 4 (severe/very severe problems). Thereby, scores of ≥2 are considered clinically significant (1, 32). All 12 ratings can be summed up to a total score, whereof a minimum number of 9 responses were strongly recommended for generating a total score (33).

### Statistical Analyses

Descriptive statistics are provided to characterize the study sample regarding demographic features and treatment-related variables (Table 1). Chi-square tests were conducted to examine the agreement between treatment response and remission status (Table 2). Distributions of PANSS and HoNOS scores (means ± standard deviations) at the time of admission and discharge are provided for the total sample as well as stratified by level of treatment response and remission status (Table 3). One-way analyses of variance (ANOVA) and Bonferroni *post hoc* group comparisons were conducted to test for group differences either at admission or at discharge. Repeated measures ANOVAs, separately for the PANSS and HoNOS, were conducted to examine whether scores changed over time, i.e., from admission to discharge. These analyses were repeated and time-by-group interaction terms were added for both levels of treatment response and remission status. In case of significant interaction effects, interaction contrasts were specified to examine group differences for change.

**Table 1:** **Overview of sample characteristics**.

		Study sample (*N* = 205)Column% / M ± SD
Sex	Male	61.0
	Female	39.0
Age		40.2 ± 12.2
Civil status	Single	66.3
	Married	19.0
	Divorced^a^	14.6
Education	No compulsory school	15.1
	Compulsory school degree	18.5
	Skilled worker	35.1
	Secondary/vocational school degree	12.7
	University degree	2.9
	N/A	15.6
Number of previous admissions to our unit	No previous admissions	59.0
	1	20.0
	2	8.3
	3 or more	12.7
Duration of treatment (in days)	≤14 days	10.2
	15–30 days	14.2
	31–60 days	28.8
	>60 days	46.8

*^a^Including N = 1 subject that was widowed*.

**Table 2:** **Agreement between level of treatment response and remission status according to the PANSS in the study sample**.

		Level of treatment response			
		NR N(%)	LR N(%)	HR N(%)	Total N(%)
**Remission status**	**NoREM**	81 (89.0)	49 (74.2)	22 (45.8)	152 (74.2)
	**REM**	10 (11.0)	17 (25.8)	26 (54.2)	53 (25.9)
	**Total**	91 (100.0)	66 (100.0)	48 (100.0)	205 (100.0)

*NR, non-response; LR, low-response; HR, high-response; NoREM, no remission; REM, remission*.

**Table 3:** **Admission and discharge PANSS and HoNOS scores for the total sample as well as subgroups of treatment response level and remission status: group comparisons and repeated measures ANOVA for change over time for the total sample and by subgroup levels**.

	PANSS			HoNOS		
Study sample	AdmissionM ± SD	Discharge M ± SD	Test for change *F*_factor(df(term),df(residual))_	AdmissionM ± SD	DischargeM ± SD	Test for change *F*_factor(df(term),df(residual))_
Total	99.7 ± 26.9	82.3 ± 26.9	*F*_time(1,204)_ = 82.34***	22.4 ± 7.3	16.0 ± 7.5	*F*_time(1,203)_ = 164.42***
**Level of treatment response**						
NR	93.2 ± 26.3	98.8 ± 27.0	*F*_time(1,202)_ = 380.79***	21.4 ± 7.7	19.1 ± 8.1	*F*_time(1,201)_ = 318.22***
LR	104.3 ± 25.6	77.6 ± 16.3	*F*_time-by-group(2,202)_ = 185.19***	23.6 ± 7.6	16.0 ± 5.9	*F*_time-by-group(2,201)_ = 53.10***
HR	105.6 ± 27.7	57.4 ± 13.2	Interaction contrasts: HR vs. NR***; LR vs. NR***; HR vs. LR***	22.8 ± 6.1	10.2 ± 4.2	Interaction contrasts: HR vs. NR***; LR vs. NR***; HR vs. LR***
*p*-Value for group differences	0.008	<0.001		0.155	<0.001	
*Post hoc* group comparisons	NR < LR, HR	HR < LR < NR		–	HR < LR < NR	
**Remission status**						
NoREM	104.8 ± 24.3	91.4 ± 24.5	*F*_time(1,203)_ = 97.81***	23.8 ± 7.1	17.9 ± 7.2	*F*_time(1,202)_ = 145.03***
REM	84.9 ± 28.7	56.3 ± 12.6	*F*_time-by-group(1,203)_ = 12.68*** Interaction contrasts: NoREM vs. REM***	18.4 ± 6.6	10.7 ± 5.5	*F*_time-by-group(1,202)_ = 2.47 n.s.
*p*-Value for group differences	<0.001	<0.001		<0.001	<0.001	

*PANSS, Positive and Negative Symptom Scales; HoNOS, Health of the Nation Outcome Scales; NR, non-response; LR, low-response; HR, high-response; NoREM, no remission; REM, remission; n.s., not significant*.

***p < 0.001.

All analyses were conducted using STATA, SE, 12th edition (34).

## Results

Our study sample consists of *N* = 205 inpatient admissions for schizophrenic psychosis (39.0% females; mean age = 40.2 ± 12.2 years; range = 17–69). The majority of the sample was single (66.3%), had finished compulsory school (18.5%), worked in a skilled job (35.1%), had their first admission to our institution (59.0%), and received treatment for more than 60 days (46.8%). For more detailed information on sample characteristics, see Table 1.

According to the degree of change in the PANSS total score from admission to discharge, *N* = 48 (23.4%) were assigned as high responders (HR), *N* = 66 (32.2%) as low responders (LR), and *N* = 91 (44.4%) as non-responders (NR) (Table 2). According to the remission criteria (18), around one-quarter (25.9%; *N* = 53) achieved remission (REM) at discharge and *N* = 152 (74.2%) did not (NoREM). The duration of treatment was significantly linked to treatment response (*F*_2,202_ = 10.29; *p* < 0.001) but not to remission status (*F*_1,203_ = 3.15; n.s.). Accordingly, the mean duration of treatment in NR was 51.7 (±42.5) days, in LR 70.3 (±44.5) days, and 88.8 (±56.1) days in HR. Pairwise group comparisons revealed HR and LR to differ significantly from NR but not from each other (not tabulated).

Despite different criteria, both level of treatment response and remission status were significantly associated (χ^2^ = 30.56; df = 2; *p* < 0.001). Accordingly, more than the half from HR also achieved REM compared to only one quarter from LR and about 11% from NR, respectively.

Table 3 displays distributions of PANSS and HoNOS scores at admission and baseline for the total sample as well as stratified by either level of treatment response or remission status.

Subsamples of either treatment response or remission status significantly differed in their initial PANSS scores at admission and at discharge (Table 3). HR had the highest baseline scores, followed by LR and, at lowest level, by NR but the difference between HR and LR was not statistically significant. At discharge, NR had the highest scores, at a similar level to those at admission; LR had lower scores than at admission; and finally, HR had the lowest scores at discharge; all differences were statistically significant. Regarding remission status, those who achieved remission at discharge had significantly lower PANSS scores at baseline and discharge than those who did not achieve remission. PANSS scores significantly decreased between admission and discharge in the total sample. However, interaction analyses revealed that this change was highly group-specific for both treatment response and remission status. Interaction contrasts indicated PANSS scores to decrease more steeply in low- and even more in HR compared to NR, as well as in REM compared to NoREM.

**Figure 1:**
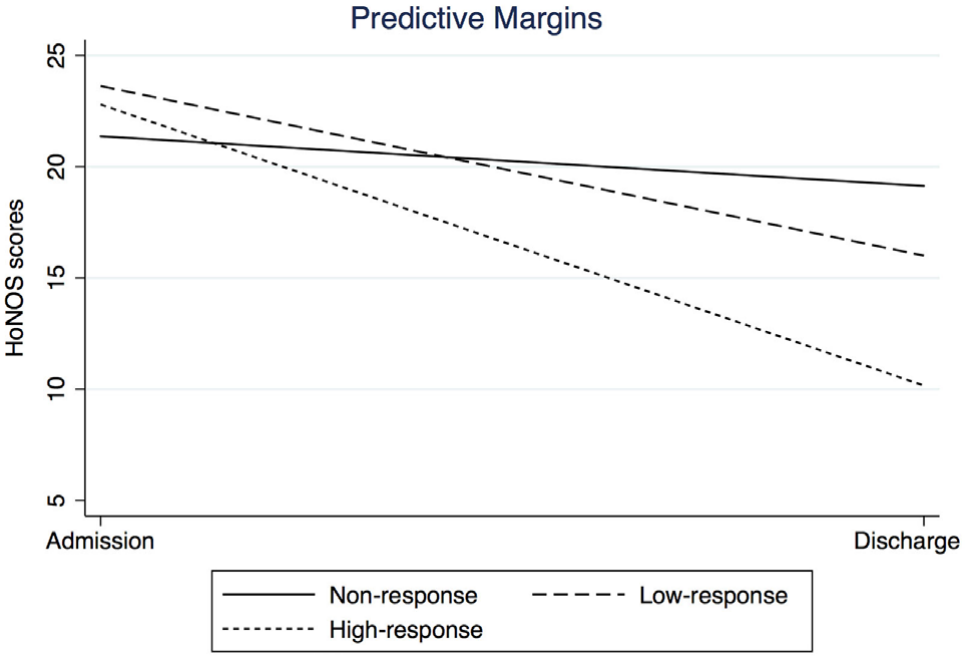
**Interaction plot: change in HoNOS between admission and discharge by level of treatment response**.

Regarding the HoNOS, no significant differences between levels of treatment response were found at admission. At discharge, all groups differed significantly from each other, with highest scores in NR followed by LR and HR with lowest scores. Overall, HoNOS scores significantly decreased over time. Interaction analyses revealed that change in HoNOS score significantly differed between treatment response groups. Contrast analyses indicated that the higher the response level (i.e., from LR to HR), the steeper the decrease was in HoNOS scores between admission and discharge (Figure 1). Finally, REM at discharge was linked to significantly lower HoNOS scores at baseline and discharge than NoREM. However, the change in HoNOS scores over time was not linked to remission status.

From demographic factors and treatment-related variables, only the duration of stay (*F*_3,200_ = 5.38; *p* = 0.001) was linked to a change in HoNOS over time (i.e., interaction with time), while demographic factors were all unrelated (not tabulated). Therefore, the duration of stay was included in the model as an additional factor to the level of treatment response but, however, revealed no additional significant interaction with time (*F*_3,198_ = 1.62; n.s.).

## Discussion

Our results revealed an overall high symptom load and functional impairment in our study sample, which supports general suggestions of a high clinical severity of schizophrenia (26). An overall symptomatic and functional improvement was apparent between admission and discharge, which far exceeded a half standard deviation, indicating a clinically relevant change (35). More than a half of the sample achieved a symptom reduction of at least 20% during their stay, while around one quarter was considered as REM on discharge from our hospital. The overall observed improvement in our study sample is in line with the reported improvement in patients with chronic schizophrenia (36, 37).

Both level of treatment response and remission status showed a certain level of agreement; nevertheless, they differed with respect to symptom severity and functioning cross-sectionally as well as over time. Although baseline functioning was comparable across levels of treatment response, those with substantial response (LR and HR) were linked to higher initial symptom severity than NR and were each related to greater improvement and better outcome at discharge than lower levels of treatment response. This observation is in line with recent findings, suggesting that those with higher initial PANSS scores tend to have a more positive outcome (38, 39).

Our findings demonstrate that baseline symptomatology largely affects symptom course over time. However, it has also been suggested that those with lower baseline severity required smaller symptom reductions to achieve a psychometrical improvement, which limits the usefulness of the change in PANSS as a primary outcome measure (28, 40). For this reason, it might be useful to accomplish outcome monitoring by standardized remission criteria in order to enhance the definition of clinical change (3, 11). Indeed, we could demonstrate that those who met the definition of remission according to RSWGcr (18) at discharge had lower total PANSS scores at discharge than non-remitters but, however, also lower baseline scores. This might explain why remitters did not show specific improvement in their functioning compared to non-remitters; it rather seems that they had lower severity and impairment already at baseline.

Finally, our findings further suggest that functional improvement over the time of treatment was indeed related to treatment duration but, however, not in addition to treatment response. This can be explained by the high association between level of treatment response and treatment duration, suggesting that there might be a functional relationship. Thus, although apparently linked to functional improvement, the length of treatment seems to become secondary in relation to symptomatic change. The missing association between the number of previous hospitalizations and subsequent functional change seems to contradict previous findings that suggested that patients with chronic schizophrenia generally tend to have poorer outcomes than first-episode patients (41), but since our data were limited to previous admissions to our institution, true effects might be underestimated or even concealed.

In sum, our findings provide strong evidence for a functional relationship between the degree of symptomatic change and the course of functioning during clinical treatment for schizophrenic psychosis. Patients with higher initial clinical severity seem to benefit more from treatment, i.e., they have more potential for clinical improvement (39). We further showed that remission as it was defined in our study did not warrant functional improvement. This would be in line with previous observations that improvement in functioning or other relevant areas sometimes occurs independently from symptom remission and is often more important for recovery than the latter (16, 42–46).

Nevertheless, this study suffers from several limitations that have to be acknowledged. First of all this study is based on a fairly small number of cases, especially in the HR group (*N* = 48). Second, our sample is based on admission data rather than on patients. Therefore, we cannot rule out that some patients may have been admitted multiple times during the study period and are therefore included more than once, even in different subsamples. Third, the required RSWGcr time criterion was not applied due to missing follow-up observations, which, however, was the usual approach in a large number of studies (47). And finally, we did not include self-report measures in the current study. Thus, we cannot conclude whether the change observed by the clinical raters was also subjectively experienced by the individuals themselves (27, 48). This, however, will be the subject of future investigations.

In conclusion, the findings of our study broadly contribute to the existing literature with regard to treatment outcome monitoring in patients with schizophrenia. We could demonstrate that higher initial clinical severity was associated with better treatment outcome. However, we showed that an improvement of psychotic symptoms, but not remission, was necessarily associated with better functioning due to different baseline severity of illness. Therefore, it appears reasonable and appropriate to use multiple scales for the assessment of clinical outcomes in specific patient groups (3, 49).

## Author Contributions

SE and MM designed the study, significantly participated in data collection, wrote the first draft of the manuscript, and significantly revised the manuscript. SV and ES conceived and designed the study, and revised the manuscript. GW conceived and designed the study, participated in data collection, and revised the manuscript. CV proof-read and significantly revised the manuscript. MM was responsible for data management and undertook all statistical analyses. All authors read and accepted the final version of the manuscript.

## Conflict of Interest Statement

The authors declare that the research was conducted in the absence of any commercial or financial relationships that could be construed as a potential conflict of interest. The reviewer SJ and handling Editor declared their shared affiliation, and the handling Editor states that the process nevertheless met the standards of a fair and objective review.
